# Moderating Effect of Pre-Exposure Prophylaxis Use on the Association Between Sexual Risk Behavior and Perceived Risk of HIV Among Brazilian Gay, Bisexual, and Other Men Who Have Sex With Men: Cross-Sectional Study

**DOI:** 10.2196/45134

**Published:** 2023-10-05

**Authors:** Kevin James Blair, Thiago S Torres, Brenda Hoagland, Daniel R B Bezerra, Valdilea G Veloso, Beatriz Grinsztejn, Jesse Clark, Paula M Luz

**Affiliations:** 1 South American Program in HIV Prevention Research Division of Infectious Diseases, Department of Medicine University of California Los Angeles Los Angeles, CA United States; 2 Department of Surgery University of California Los Angeles Los Angeles, CA United States; 3 Instituto Nacional de Infectologia Evandro Chagas Fundação Oswaldo Cruz Rio de Janeiro Brazil

**Keywords:** HIV prevention, men who have sex with men, pre-exposure prophylaxis, Latin America, risk factors, risk perception, HIV, gay, prevention, health service

## Abstract

**Background:**

Gay, bisexual, and other men who have sex with men (MSM) with a higher perceived risk of HIV are more aware of and willing to use pre-exposure prophylaxis (PrEP). PrEP is an effective HIV prevention strategy, but there is a lack of data on how PrEP use might moderate the relationship between sexual risk behavior and perceived risk of HIV. Moreover, most studies measure perceived risk of HIV via a single question.

**Objective:**

We estimated the moderating effect of PrEP use on the association between sexual risk behavior and perceived risk of HIV, measured with the 8-item Perceived Risk of HIV Scale (PRHS), among Brazilian MSM.

**Methods:**

A cross-sectional, web-based survey was completed by Brazilian Hornet app users aged ≥18 years between February and March 2020. We included data from cisgender men who reported sex with men in the previous 6 months. We evaluated the moderating effect of current PrEP use on the association between sexual risk behavior, measured via the HIV Incidence Risk Index for MSM (HIRI-MSM), and perceived risk of HIV, measured by the PRHS. Higher HIRI-MSM (range 0-45) and PRHS (range 10-40) scores indicate greater sexual behavioral risk and perceived risk of HIV, respectively. Both were standardized to *z* scores for use in multivariable linear regression models.

**Results:**

Among 4344 cisgender MSM, 448 (10.3%) were currently taking PrEP. Current PrEP users had a higher mean HIRI-MSM score (mean 21.0, SD 9.4 vs mean 13.2, SD 8.1; *P*<.001) and a lower mean PRHS score (mean 24.6, SD 5.1 vs mean 25.9, SD 4.9; *P*<.001) compared to those not currently taking PrEP. In the multivariable model, greater HIRI-MSM scores significantly predicted increased PRHS scores (β=.26, 95% CI 0.22-0.29; *P*<.001). PrEP use moderated the association between HIRI-MSM and PRHS score (interaction term β=–.30, 95% CI –0.39 to –0.21; *P*<.001), such that higher HIRI-MSM score did not predict higher PRHS score among current PrEP users.

**Conclusions:**

Our results suggest current PrEP users have confidence in PrEP’s effectiveness as an HIV prevention strategy. PrEP’s effectiveness, positive psychological impact, and the frequent HIV testing and interaction with health services required of PrEP users may jointly influence the relationship between sexual risk behavior and perceived risk of HIV among PrEP users.

## Introduction

Tenofovir-based oral pre-exposure prophylaxis (PrEP) is highly effective in preventing HIV infection in gay, bisexual, and other men who have sex with men (MSM) [1,2]. Higher perceived risk of HIV has been shown to be associated with awareness and acceptability of [3,4], interest in [5], and willingness to use PrEP [6-8] and is influential in decisions to initiate [9-13], continue or discontinue [14-17], and adhere to PrEP [18]. Taking PrEP has in turn also been shown to be associated with lower perceived risk and perceived severity of HIV [19,20] and lower sexual anxiety [21-23].

While several studies have explored the impact of PrEP use on sexual risk behaviors, such as condomless anal intercourse (CAI) [17,22,24,25], few have considered how PrEP use may moderate the relationship between sexual risk behavior and perceived risk of HIV. Potential PrEP users in the PrEP Brasil study with a higher perceived risk of HIV were more likely to say that they would discontinue condom use if they took PrEP [26]. Another study found that a higher perceived risk of HIV among PrEP users correlated with lower condom use but higher PrEP adherence [18]. A study from Amsterdam found PrEP use was associated with a lower perceived risk of HIV while sexual risk behaviors, such as CAI, were associated with a higher perceived risk, but the influence of PrEP use on the relationship between sexual risk behavior and perceived risk of HIV was not considered [19].

Most research on the perceived risk of HIV and PrEP use has measured perceived risk using a single question [5,6,18-20,27-30]. However, single question measures may be inadequate, given that perceived risk of HIV is a complex, multidimensional concept [20,27,29,31]. To address this, the 8-item Perceived Risk of HIV Scale (PRHS) was developed and subsequently validated for use in European and Brazilian Portuguese [32-34]. The PRHS covers multiple dimensions of the perceived risk of HIV, including cognitive likelihood assessment (ie, the chance of infection), intuitive assessment (ie, worry about infection), and salience of risk (ie, having thought about infection) [32].

Among Latin American countries, Brazil is a middle-income country of 203 million people and was the first country in the region to offer free national access to PrEP to eligible populations under its public health system [35,36]. As of June 2023, there were more than 64,000 PrEP users in the country, 82% of whom were MSM [13,37]. One recent study found that more than half of PrEP users in Brazil thought they had no risk of acquiring HIV, but, as with most other studies, a single question was used to measure perceived risk of HIV and the association between sexual behavior and perceived risk was not considered [38]. In this study, we used the PRHS to evaluate the moderating effect of current PrEP use on the association between sexual risk behavior and perceived risk of HIV among Brazilian MSM in the year 2020.

## Methods

### Study Design and Population

We administered a cross-sectional, web-based survey to a convenience sample of users of Hornet, a geosocial gay social network app, between February and March 2020. The survey was administered via Alchemer [39] and contained 118 questions written in Brazilian Portuguese. Requests for survey completion were sent twice and responses were collected over a 35-day period, rather than defining a sample size a priori. Additional study design details were described previously [40].

The overall study population included Hornet users ≥18 years old living in Brazil with completed surveys. We excluded those who incorrectly answered any of the 5 attention questions, which asked respondents to select a specific answer: “This question is merely a check. Please select option A from the responses below” [41]. People living with HIV were excluded. Additionally, we focused on cisgender men who reported having sex with other men in the previous 6 months, excluding those who self-identified with other genders (eg, cisgender woman, transgender man, transgender woman, or nonbinary).

### Variables

#### Sociodemographics

Age, race, sexual orientation, education level, monthly income, Brazilian state of residence, and residence in the state’s capital city metropolitan area were collected. Responses for race included White, Black, Pardo (mixed race), Indigenous, Asian (Japanese, Chinese, Korean, among others), or prefer not to respond. Income was asked in reference to the 2020 monthly minimum wage (MW) in Brazil (BRL1039, ~US $190), which was grouped into low-income (no salary, 1-2× MW), middle-income (2-6× MW), and high-income (>6× MW).

#### Sexual Health and Behavior

We asked about the timing of the most recent HIV test, which was categorized as within the last 6 months, more than 6 months ago, or never tested. Knowledge about HIV was measured via the HIV/AIDS Knowledge Assessment (HIV-KA) tool, which is scored 0 to 12, with higher scores indicating greater HIV knowledge [40,42,43]. Respondents indicated whether they had a steady partner and, if so, their steady partner’s HIV status. We also asked about each of the following over the previous 6 months: number of male sex partners, any male sex partners known to be living with HIV, receptive CAI, sexually transmitted infection (STI) diagnoses (syphilis, urethral or rectal gonorrhea, urethral or rectal chlamydia), transactional sex (sex for money or other good), or chemsex (see Table 2 footnote).

Level of engagement in sexual risk behaviors was estimated via the HIV Incidence Risk Index for MSM (HIRI-MSM) score, calculated based on respondent age and the following behaviors with men in the previous 6 months: number of sex partners, number of sex partners living with HIV, receptive CAI with any HIV-status partner, insertive CAI with a partner living with HIV, and use of stimulants (cocaine; crack, pasta básica, or oxy; ecstasy; methamphetamines; gamma hydroxybutyrate or gamma butyrolactone; poppers; or other inhalants) [44,45]. HIRI-MSM is scored from 0 to 45, and a score of >10 has been considered an indication that the respondents may benefit from PrEP.

#### PrEP Use

We asked respondents if they had ever heard of PrEP (awareness), and those who responded affirmatively were asked about PrEP use via the question “Are you taking or have you taken PrEP?” Options included “No, I have never taken PrEP,” “Yes, I took PrEP but stopped,” or “Yes, I am currently taking PrEP.” Respondents who were not aware of PrEP were grouped with never PrEP users. For this analysis, never and past PrEP use were grouped together as “no current PrEP use.” To evaluate the robustness of the results from the regression models, a sensitivity analysis was performed in which we excluded past PrEP users from the regression models. Current PrEP users were asked about adherence based on the number of days they had taken PrEP over the previous week, categorized as every day, 4 to 6 days, or 3 or fewer days.

#### Outcome

Perceived risk of HIV was measured via the PRHS [32,34]. The PRHS consists of 8 Likert-scale questions covering different dimensions of perceived risk, as described in the introduction. Prior work showed the scale to be valid for use among MSM in Brazil [34]. Total scores range from 10 to 40, with higher scores indicating greater perceived risk of HIV.

### Statistical Analyses

The outcome of this analysis was the PRHS score, and our primary aim was to assess the effect of the HIRI-MSM score and current PrEP use as explanatory variables as well as their potential interaction. All analyses were performed using R Software for Statistical Computing (version 4.0.3; R Core Team). Categorical variables were described using proportions and continuous variables using mean with SD and median with IQR. Cronbach α was used to assess the internal reliability of the PRHS and the HIV-KA tool [46]. We present and compare mean PRHS scores across various demographic, sexual health, and sexual risk behavior variables using the Student *t* test (2 variable categories) or ANOVA *F* test (3 or more categories). The comparison of PRHS scores across sexual risk behaviors was stratified according to current versus no current PrEP use.

We used multivariable linear regression models to estimate the association between HIRI-MSM score and PRHS score and the moderating effect of current PrEP use on that association. Due to the small sample size for individual variable categories, respondents with missing data and those who identified their race as Asian or Indigenous were excluded from the regression model. For inclusion in regression models, PRHS, HIRI-MSM, and HIV-KA scores were standardized to *z* scores by subtracting the mean overall score from each participant’s score and dividing the difference by the overall SD. Multivariable models were created by first including HIRI-MSM as the explanatory variable, followed by current PrEP use, and then an interaction term between the two. Finally, a full multivariable model included variables for which prior work had shown an association with perceived risk of HIV, including race, education, Brazilian state, sexual orientation, HIV knowledge, steady partner, transactional sex, and timing of last HIV test [4,19,22,27,30,34,47-52]. Age was not included as a covariate in the final model since it is accounted for in the HIRI-MSM score. We verified the fitted model’s assumptions using standard diagnostic plots and observed no violations. To aid in the visualization of the moderating effect of PrEP, we present a graphical representation of the interaction between the HIRI-MSM score and PrEP use created using the “effects” package in R [53].

### Ethics Approval

This study received approval from the human subjects ethics committee at Instituto Nacional de Infectologia Evandro Chagas of Fundação Oswaldo Cruz (#CAAE 01777918.0.0000.5262) and was exempt from review by the University of California, Los Angeles institutional review board. All participants provided electronic informed consent before survey initiation.

## Results

**Figure 1:**
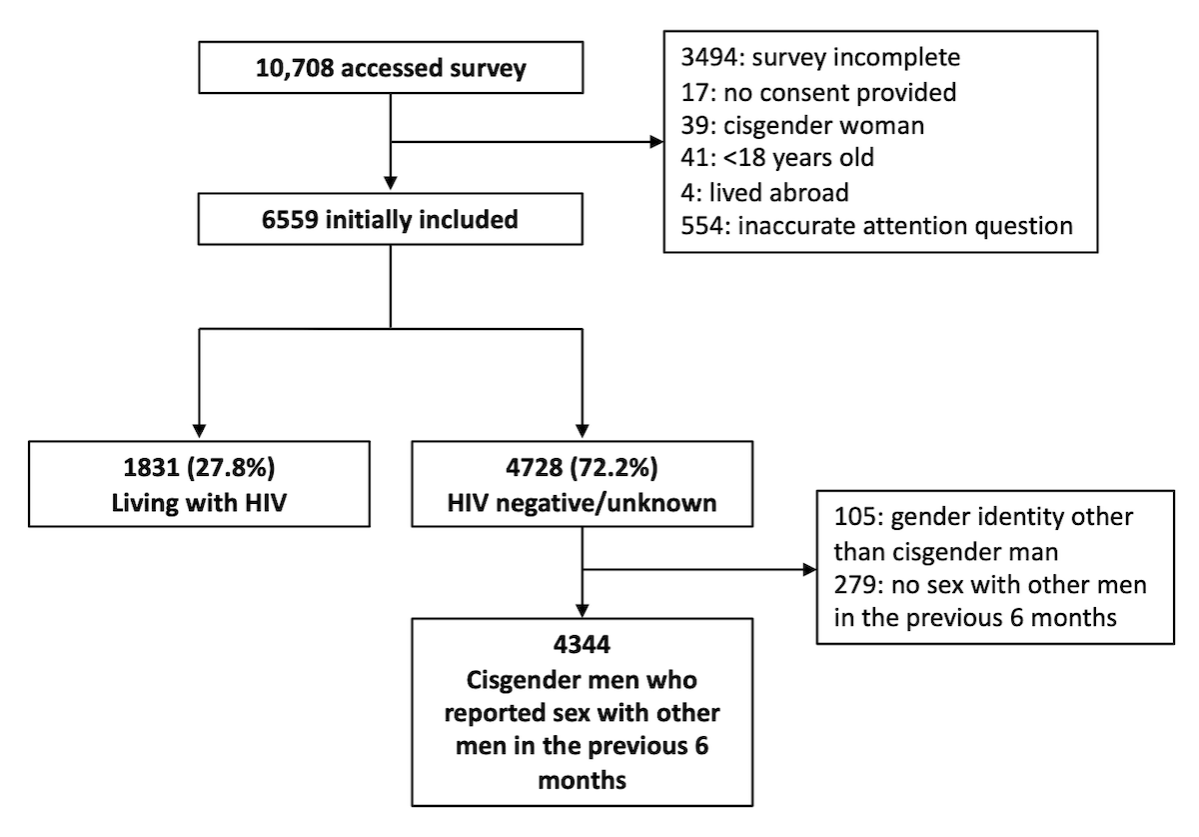
Flow diagram for study inclusion.

A total of 4344 cisgender MSM who reported sex with other men in the previous 6 months were included (Figure 1). The overall sample had a mean age of 34.4 (SD 10.2) years (Table 1). Most reported White race (n=2624, 60.4%), identified as gay or homosexual (n=3643, 83.9%), and had a university or higher-level education (n=2992, 68.9%). Most respondents lived in the Southeast region of Brazil, with the Brazilian states of São Paulo (n=2284, 52.6%) and Rio de Janeiro (n=919, 21.2%) being the most common. More than two-thirds lived in their state’s capital metropolitan area (n=2963, 68.2%). The majority (n=2321, 53.4%) of respondents had tested for HIV within the previous 6 months, and knowledge about HIV prevention was high (median HIV-KA score 11, IQR 10-12; α=.61). Nearly all (n=3996, 92%) were aware of PrEP, and 448 (10.3%) were currently taking PrEP.

The PRHS had high internal reliability (α=.75). Mean overall PRHS score was 25.8 (SD 4.9). The mean PRHS score was higher among gay-identifying MSM compared to other sexual orientations (26 vs 24.9; *P*<.001) and among university-educated respondents compared to those with secondary- or lower-level education (26 vs 25.4; *P*<.001). There was no significant difference in PRHS score across age, race, income, or Brazilian state. PRHS scores were higher among those who were aware of PrEP, as compared to those not aware (25.9 vs 24.3; *P*<.001), but were lower among MSM currently taking, compared to not currently taking PrEP (24.6 vs 25.9; *P*<.001).

Current PrEP users had significantly higher mean HIRI-MSM scores compared to MSM not currently taking PrEP (21.0 vs 13.2; *P*<.001) (Table 2). Among MSM not currently taking PrEP, higher PRHS scores significantly correlated with reporting a greater number of sex partners (*P*<.001), a steady partner living with HIV or with unknown HIV status (*P*<.001), any sex partner living with HIV (*P*<.001), receptive CAI (*P*<.001), STI diagnoses (*P*<.001), chemsex (*P*<.001), and transactional sex (*P*<.001). Conversely, among MSM currently taking PrEP, the only sexual risk behavior significantly associated with higher PRHS score was having a steady partner living with HIV or with unknown HIV status (*P*=.03). Among MSM currently taking PrEP, having taken PrEP every day in the previous week, as compared to 3 or fewer days, was associated with a lower mean PRHS score (24.4 vs 27.2; *P*=.05).

**Figure 2:**
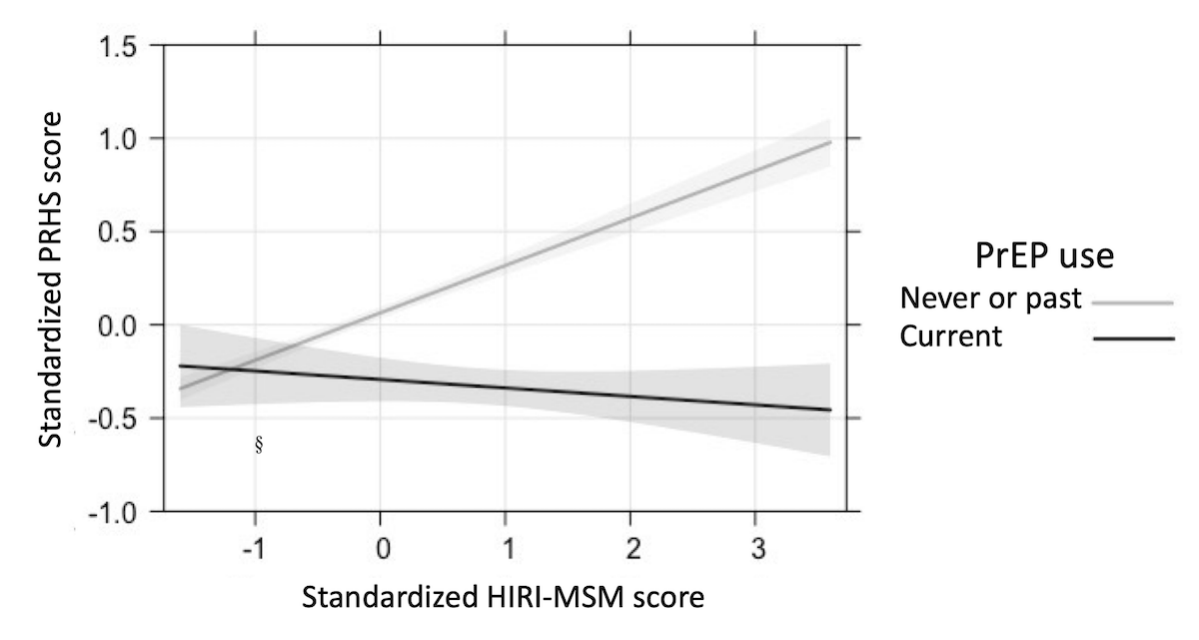
Graphical representation of the joint effect of sexual risk behavior (measured via HIRI-MSM) and current PrEP use on perceived risk of HIV (measured via PRHS). The final model was adjusted for race, education, Brazilian state, sexual orientation, HIV knowledge, steady partner, transactional sex, and timing of last HIV test. HIRI-MSM: HIV Incidence Risk Index for men who have sex with men; PrEP: pre-exposure prophylaxis; PRHS: Perceived Risk of HIV Scale.

Higher HIRI-MSM scores significantly predicted higher PRHS scores in bivariate linear regression (model 1: β=.19, 95% CI 0.16-0.22; *P*<.001), meaning a 0.19 higher standardized PRHS score per 1 SD increase in standardized HIRI-MSM score (Table 3). The magnitude of the β coefficient for the HIRI-MSM score increased when PrEP use was included in the model (model 2: β=.23, 95% CI 0.20-0.26; *P*<.001), and when the HIRI:PrEP interaction term was added as an explanatory variable (model 3: β=.27, 95% CI 0.24-0.30; *P*<.001). In the fully adjusted model, standardized HIRI-MSM score had a positive association (β=.26, 95% CI 0.22-0.29; *P*<.001) and current PrEP use had a significant negative association (β=–.36, 95% CI –0.48 to –0.23; *P*<.001) with standardized PRHS scores. The estimated β coefficient for the HIRI-MSM:PrEP use interaction term indicated a significant moderating effect on the association between HIRI-MSM and PRHS score by current PrEP use (β=–0.30, 95% CI –0.39 to –0.21; *P*<.001). Results were unchanged when past PrEP users were excluded from regression models (Table S1 in Multimedia Appendix 1). The graphical representation of HIRI-MSM:PrEP use interaction shows how among those not using PrEP, higher PRHS scores were associated with higher HIRI-MSM scores. In contrast, among those taking PrEP, PRHS scores were unchanged by higher HIRI-MSM scores (Figure 2).

Among covariates in the full adjusted regression model (Table 3), having a steady partner who was HIV-negative compared to no steady partner was associated with a lower PRHS score (β=–.24, 95% CI –0.31 to –0.18; *P*<.001). Conversely, having a university or higher-level education compared to secondary- or lower-level (β=.12, 95% CI 0.06 to 0.19; *P*<.001), increasing HIV-KA score (β=.12, 95% CI 0.09 to 0.15; *P*<.001), identifying as gay compared to other sexual orientations (β=.11, 95% CI 0.03 to 0.19; *P*=.007), having a partner living with HIV or with unknown HIV status (β=.16, 95% CI 0.05 to 0.28; *P*=.004), and testing for HIV more than 6 months ago (β=.08, 95% CI 0.01 to 0.15; *P*=.02) were all associated with higher PRHS scores.

**Table 1:** Mean Perceived Risk of HIV Scale scores compared across demographic and select sexual health characteristics from a cross-sectional sample of Brazilian men who have sex with men in 2020.

Variables			Total, N=4344; n (%)	PRHS^a^ score; mean (SD)		*P* value	
**Age group (years)**							.58
	18-24	644 (14.8)		25.6 (4.9)			
	25-29	955 (22)		25.9 (4.8)			
	30-39	1595 (36.7)		25.9 (4.9)			
	≥40	1150 (26.5)		25.7 (4.9)			
	Mean (SD)	34.4 (10.2)		N/A^b^	N/A		
**Race**							.48
	White	2,624 (60.4)		25.8 (4.8)			
	Pardo	1112 (25.6)		25.8 (4.9)			
	Black	406 (9.3)		26 (5)			
	Asian	70 (1.6)		25.3 (4.6)			
	Indigenous	30 (0.7)		24.3 (5.9)			
	Unanswered	102 (2.3)		26.1 (5.3)			
**Sexual orientation**							<.001
	Gay or homosexual	3643 (83.9)		26 (4.9)			
	Other^c^	701 (16.1)		24.9 (4.8)			
**Education level^d^**							<.001
	University or higher	2992 (68.9)		26 (4.9)			
	Secondary or less	1276 (29.4)		25.4 (4.9)			
	Unanswered	76 (1.7)		26.3 (5.2)			
**Income level^e^**							.39
	High	1189 (27.4)		25.9 (4.9)			
	Middle	1977 (45.5)		25.8 (4.8)			
	Low	1178 (27.1)		25.6 (5.1)			
**Brazilian state**							.44
	São Paulo	2284 (52.6)		25.8 (4.8)			
	Rio de Janeiro	919 (21.2)		26 (5.1)			
	Other	1141 (26.3)		25.7 (4.9)			
**Live in state’s capital metropolitan area**							.70
	Yes	2963 (68.2)		25.8 (4.9)			
	No	1381 (31.8)		25.7 (4.9)			
**Timing of most recent HIV test**							.06
	Within the last 6 months	2321 (53.4)		25.7 (4.9)			
	More than 6 months ago	1522 (35)		26 (4.8)			
	Never tested	431 (9.9)		25.4 (4.8)			
	Unanswered	70 (1.6)		25.9 (6.2)			
**HIV-KA^f^ score (range 0-12)**							N/A
	Mean (SD)	10.7 (1.6)		N/A	N/A		
	Median (IQR)	11 (10-12)		N/A	N/A		
**PrEP^g^ Awareness**							<.001
	Yes	3996 (92)		25.9 (4.8)			
	No	348 (8)		24.3 (5.3)			
**PrEP use**							<.001
	Current	448 (10.3)		24.6 (5.1)			
	No current	3896 (89.7)		25.9 (4.9)			
	Past^h^	156 (3.6)		26.6 (4.9)			
	Never^h^	3740 (86.1)		25.9 (4.9)			

^a^PRHS: Perceived Risk of HIV Scale.

^b^N/A: not applicable.

^c^“Other” includes responses of heterosexual, bisexual, pansexual, asexual, and other.

^d^Education levels of college and postgraduate were combined into “university or higher”; secondary, primary, or less than primary were combined into “secondary or less.”

^e^We grouped no salary, 1×, and 2× the minimum wage as “low-income,” 2-6× as “middle-income,” and >6× as “high-income.” Monthly minimum monthly wage in 2020 was BRL1039 (~US $190).

^f^HIV-KA: HIV/AIDS Knowledge Assessment; high scores indicate higher level of HIV knowledge.

^g^PrEP: pre-exposure prophylaxis.

^h^Past and never PrEP use were combined as no current PrEP use for further analyses.

**Table 2:** Mean Perceived Risk of HIV Scale scores compared across sexual risk behaviors and stratified by current versus no current pre-exposure prophylaxis use.

Variable		Current PrEP^a^ use, n=448									No current PrEP use, n=3896					
		Respondents, n (%)		PRHS^b^ score, mean (SD)		*P* value			Respondents, n (%)				PRHS score, mean (SD)		*P* value	
**HIRI-MSM^c^ score**							.82									<.001
	Mean (SD)	21 (9.4)	N/A^d^					13.2 (8.1)				N/A				
	>10	387 (86.4)	24.5 (5.1)					2309 (59.3)				26.8 (4.7)				
	<10	61 (13.6)	24.7 (5.1)					1587 (40.7)				24.7 (4.8)				
**Number of sex partners^e^**							.57									<.001
	1-5	111 (24.8)	25 (4.9)					2196 (56.4)				25.1 (4.8)				
	6-10	105 (23.4)	24.4 (4.9)					818 (21)				26.5 (4.6)				
	11-30	138 (30.8)	24.7 (5.4)					637 (16.4)				27.2 (4.5)				
	30+	94 (21)	24 (5)					245 (6.3)				28.3 (5.4)				
**Steady partner^e^**							.03									<.001
	No	305 (68.1)	24.5 (4.9)					2603 (66.8)				26.2 (4.8)				
	Yes, HIV negative	96 (21.4)	23.8 (5.3)					1019 (26.2)				25 (4.9)				
	Yes, living with HIV	33 (7.4)	26 (4.8)					120 (3.1)				27.4 (4.9)				
	Yes, I don’t know their HIV status	14 (3.1)	27.2 (6.8)					154 (4)				27 (4.6)				
**Sex partner living with HIV^e^**							.40									<.001
	Yes	155 (34.6)	24.8 (5.3)					346 (8.9)				27.8 (4.8)				
	No	293 (65.4)	24.4 (5)					3550 (91.1)				25.7 (4.8)				
**Receptive CAI^e,f^**							.67									<.001
	Yes	294 (65.6)	24.6 (5.1)					1558 (40)				27 (4.7)				
	No	154 (34.4)	24.4 (5.1)					2338 (60)				25.2 (4.8)				
**STI^g^ diagnosis^e^**							.88									<.001
	Yes	128 (28.6)	24.5 (5.3)					452 (11.6)				27.8 (4.8)				
	No or unknown	320 (71.4)	24.6 (5)					3444 (88.4)				25.7 (4.8)				
**Chemsex^e,h^**							.91									<.001
	Yes	168 (37.5)	24.5 (5.1)					837 (21.5)				27.2 (4.8)				
	No	280 (62.5)	24.6 (5.1)					3059 (78.5)				25.6 (4.8)				
**Transactional sex^i^**							.09									.001
	Yes	33 (7.4)	23.1 (5)					242 (6.2)				26.9 (5.7)				
	No	415 (92.6)	24.7 (5.1)					3654 (93.8)				25.9 (4.8)				
**PrEP adherence in past week^j^**							.05									N/A
	Every day	393 (87.7)	24.4 (5.1)					N/A				N/A				
	4 to 6 days	39 (8.7)	25.4 (4.4)					N/A				N/A				
	3 or fewer days	16 (3.6)	27.2 (6.3)					N/A				N/A				

^a^PrEP: pre-exposure prophylaxis.

^b^PRHS: Perceived Risk of HIV Scale.

^c^HIRI-MSM: HIV Incidence Risk Index for Men Who Have Sex With Men. The score is calculated based on respondent age and the following behaviors in the previous 6 months: number of male partners, number of male partners living with HIV, receptive condomless anal sex with any HIV-status partner, insertive condomless anal sex with a partner living with HIV, use of stimulants (cocaine; crack, basic paste, or oxy; ecstasy; methamphetamines [crystal or speed]; gamma hydroxybutyrate or gamma butyrolactone; poppers; or other inhalants). A score of >10 suggests the respondent may benefit from pre-exposure prophylaxis.

^d^N/A: not applicable.

^e^Asked in reference to the previous 6 months.

^f^CAI: condomless anal intercourse.

^g^STI: sexually transmitted infection. The survey specified syphilis, urethral or rectal gonorrhea, and urethral or rectal chlamydia.

^h^Use of any of the following substances before or during sex: cocaine; crack, basic paste, or oxy; marijuana, hashish, or skank; ecstasy; methamphetamines (crystal or speed); gamma hydroxybutyrate or gamma butyrolactone; poppers; other inhalants; mephedrone; hallucinogens (LSD, mushroom tea, others); or others.

^i^Sex for money or some other good (eg, gifts, housing).

^j^Answered only by those currently taking PrEP (n=448).

**Table 3:** Linear regression models demonstrating the moderating effect of current PrEP use on the association between standardized PRHS and HIRI-MSM scores (n=4092).^a^

Variable			Model 1				Model 2				Model 3				Full model		
			β (95% CI)		*P* value		β (95% CI)		*P* value		β (95% CI)		*P* value		β (95% CI)		*P* value
Intercept			0 (–0.03 to 0.03)		0.99		.05 (0.02 to 0.08)		.001		.06 (0.02 to 0.09)		<.001		–.11 (–0.21 to –0.01)		.04
HIRI-MSM^b^ score, standardized			.19 (0.16 to 0.22)		<.001		.23 (0.20 to 0.26)		<.001		.27 (0.24 to 0.30)		<.001		.26 (0.22 to 0.29)		<.001
Current PrEP^c^ use			N/A^d^		N/A		–.52 (–0.62 to –0.41)		<.001		–.31 (–0.43 to –0.19)		<.001		–.36 (–0.48 to –0.23)		<.001
HIRI:PrEP interaction			N/A		N/A		N/A		N/A		–.30 (–0.39 to –0.21)		<.001		–.30 (–0.39 to –0.21)		<.001
**Race**																	
	Black^e^	N/A		N/A		N/A		N/A		N/A		N/A		.04 (–0.07 to 0.14)		.49	
	Pardo^e^	N/A		N/A		N/A		N/A		N/A		N/A		.02 (–0.05 to 0.09)		.61	
**Education**																	
	University or higher^e^	N/A		N/A		N/A		N/A		N/A		N/A		.12 (0.06 to 0.19)		<.001	
**State**																	
	Rio de Janeiro^e^	N/A		N/A		N/A		N/A		N/A		N/A		.03 (–0.05 to 0.10)		.48	
	Other^e^	N/A		N/A		N/A		N/A		N/A		N/A		–.01 (–0.08 to 0.06)		.73	
**Sexual orientation**																	
	Gay^e^	N/A		N/A		N/A		N/A		N/A		N/A		.11 (0.03 to 0.19)		.007	
HIV-KA^f^ score, standardized			N/A		N/A		N/A		N/A		N/A		N/A		.12 (0.09 to 0.15)		<.001
**Steady partner**																	
	HIV-negative^e^	N/A		N/A		N/A		N/A		N/A		N/A		–.24 (–0.31 to –0.18)		<.001	
	Living with HIV or HIV-unknown^e^	N/A		N/A		N/A		N/A		N/A		N/A		.16 (0.05 to 0.28)		.004	
Transactional sex^e^			N/A		N/A		N/A		N/A		N/A		N/A		.11 (–0.01 to 0.23)		.073
**Last HIV test**																	
	6 or more months ago^e^	N/A		N/A		N/A		N/A		N/A		N/A		.08 (0.01 to 0.15)		.016	
	Never^e^	N/A		N/A		N/A		N/A		N/A		N/A		–.06 (–0.17 to 0.04)		.24	
Adjusted *R*^2^			0.034		N/A		0.057		N/A		0.066		N/A		0.103		N/A

^a^Given small sample sizes, Asian (n=70), Indigenous (n=30), and respondents with missing data (n=152) were excluded.

^b^HIRI-MSM: HIV Incidence Risk Index-men who have sex with men.

^c^PrEP: pre-exposure prophylaxis.

^d^N/A: not applicable.

^e^Reference categories are as follows: race: White; education: secondary or lower; Brazilian state: São Paulo; sexual orientation: other; steady partner: no steady partner; transactional sex: no; last HIV test: 6 months or less.

^f^HIV-KA: HIV/AIDS Knowledge Assessment.

## Discussion

### Principal Findings

We found that current PrEP use among MSM in Brazil had a significant moderating effect on the association between sexual risk behavior and perceived risk of HIV. While there was an overall positive association between increasing HIRI-MSM and PRHS scores, the negative moderating effect of PrEP use resulted in no significant association between HIRI-MSM and PRHS scores among current PrEP users. Perceived risk of HIV was significantly positively associated with all sexual risk behavior variables among MSM not currently taking PrEP, but not among MSM currently taking PrEP.

One explanation for our findings is that Brazilian MSM who were taking PrEP were appropriately confident and optimistic about its effectiveness as an HIV prevention strategy. This understanding has been referred to as prevention optimism, which Holt and Murphy [54] define as “the belief that it is easier to avoid HIV infection or transmission because of PrEP and that it is more acceptable and safer to engage in condomless sex because the risk of HIV is reduced.” Prevention optimism could be a key mediator between sexual risk behavior and perceived risk of HIV and may be a useful adjunct to interpreting cross-sectional data [31,54]. While we did not specifically assess prevention optimism, future research could consider assessing agreement with statements such as “It is safe for me to have sex without condoms if I am using PrEP” [54]. There is a concern that the lower perceived risk of HIV among PrEP users may indirectly increase the risk of bacterial STIs via increased sexual risk behaviors, such as number of sexual partners and CAI [55], though data on the temporal relationship between PrEP use and changes in sexual behavior and STI acquisition are mixed [22,56,57]. Nevertheless, the lack of association between sexual risk behaviors and perceived risk of HIV among PrEP users in our study highlights the importance of including the prevention of bacterial STIs as a part of PrEP implementation policy. Other future considerations include the adoption of prophylaxis options for bacterial STIs to be taken by PrEP users [58].

Another important factor influencing the relationship between sexual risk behavior and perceived risk of HIV is the positive psychological impact of PrEP, which has been shown to be associated with reduced anxiety and fear related to sexual intercourse, and relatedly, increased sexual pleasure and intimacy [21-23,59-61]. PrEP use allows some MSM to engage in sexual behaviors that were previously the cause of anxiety and may facilitate connectedness within the MSM community [21,23]. Moreover, the ability of MSM taking PrEP to forgo condoms without worry about acquiring HIV can have a significant positive impact on sexual pleasure and satisfaction. In our study, we used a validated scale to capture PrEP’s impact on the multiple dimensions of the perceived risk of HIV, but future studies could more specifically consider the positive mental health impact of PrEP use among Brazilian MSM.

The frequent interaction with the health care system required of those taking PrEP may also influence an individual’s perceived risk of HIV and offer an opportunity for counseling interventions focused on reducing risk of bacterial STIs among PrEP users [4,22,56]. In Brazil, MSM receiving PrEP from the public health system must have appointments and HIV testing with their sexual health care provider every 3-6 months [62]. Regular HIV testing may contribute to lower perceived risk, since we found that those who had last tested for HIV within the previous 6 months had lower perceived risk of HIV. Additionally, the counseling provided at these visits could influence perceived risk by way of an improved understanding of how one’s sexual behaviors relate to HIV risk and how PrEP may mitigate that risk [31]. Jaspal et al [4] proposed that perceived risk of HIV may mediate the pathway between HIV knowledge and sexual behavior. We previously found that PrEP use was associated with greater HIV knowledge among Brazilian MSM [40], and in this study we found greater HIV knowledge was associated with greater perceived risk of HIV among our overall sample. However, we did not explore the association among current PrEP users specifically, and future studies could compare the association between HIV knowledge and perceived risk of HIV stratified by PrEP use.

Like previous work from Brazil [47], we found higher education and reporting one’s sexual orientation as gay to be associated with a higher perceived risk of HIV, whereas having a steady HIV-negative partner, compared to no steady partner, was associated with a lower perceived risk of HIV. Having tested for HIV more than 6 months ago, compared to within 6 months, was associated with a higher perceived risk of HIV among our sample, but findings from other studies have been mixed. MSM in the United States who had tested for HIV more than 1 year ago, compared to within the past year, were found to have lower perceived risk of HIV [27]. Another multicountry study found that as the years since the last HIV test increased, the perceived risk of HIV decreased among MSM in the United Kingdom but increased among MSM in Thailand [51].

Our use of the PRHS is unique among similar studies. Much of the prior research on perceived risk of HIV and PrEP use has measured perceived risk using agreement with a single Likert scale statement [6,18-20,27] or a single question with categorical responses of low, medium, and high risk [5,28-30]. The PRHS, in contrast, offers a more robust measure by assessing multiple dimensions of the perceived risk of HIV [32].

It is important to acknowledge that PrEP users are not a monolith, and the distribution of PRHS scores among PrEP users in our study demonstrated a range from low to high perceived risk of HIV. While we have demonstrated that PrEP use, on average, has a moderating effect on the relationship between sexual risk behavior and perceived risk of HIV, we did not study how this association may vary based on the type of PrEP user, and we did not have longitudinal data to assess how risk perception may change over time. Previous research has shown that PrEP users may fall into distinct groups depending on their perceived risk, sexual risk behaviors, and use of other prevention methods. A longitudinal study from France and Canada found that participants enrolled in the PrEP trial fell into distinct risk perception trajectories throughout the study follow-up, which they described as low-, medium-, and high perceived risk [18]. Similarly, a discrete choice experiment conducted among MSM in Singapore found that respondents fell into 3 different groups based on PrEP and condom preferences, and their perceived risk of HIV and STIs [63]. MSM PrEP-users from Australia fell into 4 distinct groupings based on a latent class analysis of risk behaviors and perceived risk of STIs, with highly variable views toward STI risk among the 4 groups [64]. Understanding the potential heterogeneity of PrEP users is critical in the implementation of future PrEP initiatives in Latin America and when considering interventions to reduce the risk of bacterial STIs among PrEP users.

Our study has several limitations. The cross-sectional nature of these data limited our ability to assert temporality between exposures of interest and perceived risk of HIV. These data were self-reported and may have been subject to recall or social desirability bias. We focused on cisgender MSM because of the small sample size of respondents reporting other gender identities; additional research is needed to characterize how PrEP use relates to sexual behavior and perceived risk of HIV among transgender, nonbinary, and gender-diverse individuals in Brazil. The majority of MSM in our sample were from either São Paulo or Rio de Janeiro, which may limit the generalizability of our findings, since previous research showed that perceived risk of HIV risk varies by region of Brazil [52]. Our findings are also specific to MSM who use Hornet and may not be generalizable to those who use other social networking app or those who do not have access. Additionally, the 10.3% of our sample who were taking PrEP at the time of this study may represent early adopters of PrEP and may not necessarily be representative of the population of MSM who will use PrEP as availability expands. The HIRI-MSM tool is a proxy measure of behavior risk of HIV, and other measures of sexual behavior may have different associations with perceived risk. Finally, the HIRI-MSM tool was developed for use in the United States and has not been specifically validated for use among Brazilian MSM, but we chose it because we preferred a continuous rather than dichotomous explanatory variable and because it has widespread familiarity among the HIV prevention research community and previous use among Brazilian MSM [52].

### Conclusions

PrEP is highly effective at preventing HIV acquisition, and its use among MSM is associated with lower perceived risk of HIV compared to those not taking PrEP. We found that while higher HIRI-MSM scores were predictive of higher PRHS scores among our overall sample, the association was moderated by PrEP use, resulting in no significant association between sexual risk behavior and perceived risk of HIV among current PrEP users in Brazil. PrEP’s objective efficacy, positive psychological impact, and the frequent HIV testing and interaction with the health care system required of PrEP users may jointly influence the relationship between sexual risk behavior and perceived risk of HIV, though additional research is needed to measure how each of these factors influences the moderating effect of PrEP. Future studies should explore the concept of prevention optimism and consider the temporal associations between PrEP use, sexual risk behaviors, and perceived HIV risk. Finally, the expansion of PrEP access in the Latin American region should consider how the lower perceived risk of HIV among PrEP users may necessitate targeted counseling on the risk of bacterial STIs as well as adjunct STI prevention modalities.

## Appendix

Multimedia Appendix 1 Linear regression models demonstrating the moderating effect of current PrEP use on the association between standardized PRHS and HIRI-MSM scores, excluding past PrEP users N=3946.

## Abbreviations

CAI: condomless anal intercourse
HIRI-MSM: HIV Incidence Risk Index for men who have sex with men
HIV-KA: HIV/AIDS Knowledge Assessment
MSM: men who have sex with men
MW: minimum wage
NIMH: National Institute of Mental Health
PrEP: pre-exposure prophylaxis
PRHS: Perceived Risk of HIV Scale
STI: sexually transmitted infection

## Data Availability

The data set analyzed during this study is available from the corresponding author on reasonable request.
