# Supplementary material for: Moderating Effect of Pre-Exposure Prophylaxis Use on the Association Between Sexual Risk Behavior and Perceived Risk of HIV Among Brazilian Gay, Bisexual, and Other Men Who Have Sex With Men: Cross-Sectional Study
Source: JMIR Public Health Surveill. 2023 Oct 5;9:e45134. doi: 10.2196/45134 (PMC10587815; doi:10.2196/45134)
Supplement: Multimedia Appendix 1 [file publichealth_v9i1e45134_app1.docx]

**Table S1.** Linear regression models demonstrating the moderating effect of current PrEP use on the association between standardized PRHS and HIRI-MSM scores, excluding past PrEP users N=3946.^a^

| Variable | Model 1 |  | Model 2 |  | Model 3 |  | Full Model |  |
| --- | --- | --- | --- | --- | --- | --- | --- | --- |
|  | β [95% CI] | *P* value | β (95% CI) | *P* value | β (95% CI) | *P* value | β (95% CI) | *P* value |
| (Intercept) | 0.00 [-0.03­ to 0.03] | 1.000 | 0.05 [0.02 to 0.09] | .001 | 0.06 [0.03 to 0.09] | <.001 | -0.11 [-0.21 to -0.001] | .047 |
| HIRI-MSM score, standardized | 0.18 [0.15 to 0.21] | <.001 | 0.23 [0.19 to 0.26] | <.001 | 0.27 [0.23 to 0.30] | <.001 | 0.25 [0.22 to 0.29] | <.001 |
| Current PrEP Use ^b^ |  |  | -0.51 [-0.61 to -0.41] | <.001 | -0.30 [-0.42 to -0.18] | <.001 | -0.36 [-0.48 to -0.23] | <.001 |
| HIRI:PrEP Interaction |  |  |  |  | -0.30 [-0.39 to -0.21] | <.001 | -0.30 [-0.39 to -0.21] | <.001 |
| Race: Black ^c^ |  |  |  |  |  |  | 0.04 [-0.06 to 0.14] | .450 |
| Race: *Pardo* ^c^ |  |  |  |  |  |  | 0.02 [-0.05 to 0.09] | .648 |
| Education: University or higher ^c^ |  |  |  |  |  |  | 0.13 [0.07 to 0.20] | <.001 |
| State: Rio de Janeiro ^c^ |  |  |  |  |  |  | 0.02 [-0.05 to 0.10] | .531 |
| State: Other ^c^ |  |  |  |  |  |  | -0.01 [-0.08 to 0.06] | .848 |
| Sexual Orientation: Gay ^c^ |  |  |  |  |  |  | 0.10 [0.02 to 0.18] | .013 |
| HIV-KA Score, standardized |  |  |  |  |  |  | 0.12 [0.09 to 0.15] | <.001 |
| Steady partner, HIV-negative ^c^ |  |  |  |  |  |  | -0.25 [-0.32 to -0.18] | <.001 |
| Steady partner, living with HIV or HIV-unknown ^c^ |  |  |  |  |  |  | 0.20 [0.08 to 0.32] | <.001 |
| Transactional Sex ^b^ |  |  |  |  |  |  | 0.12 [-0.001 to 0.25] | .052 |
| Last HIV Test: six or more months ago ^c^ |  |  |  |  |  |  | 0.07 [0.01 to 0.14] | .033 |
| Last HIV Test: Never ^c^ |  |  |  |  |  |  | -0.07 [-0.18 to 0.03] | .183 |
| Adjusted *R^2^* | 0.032 |  | 0.055 |  | 0.064 |  | 0.102 |  |

^a^ Sensitivity analysis excluding past PrEP users (n=142) from the linear regression models

^b^ Reference category “No”

^c^ Reference categories are as follows: Race: White; Education: Secondary or lower; Brazilian state: São Paulo; Sexual Orientation: Other; Steady partner: no steady partner; Last HIV Test: 6 months or less

Abbreviations: HIV Incidence Risk Index (HIRI), Perceived Risk of HIV Scale (PRHS), PrEP-Exposure Prophylaxis (PrEP), Human Immunodeficiency Virus (HIV), HIV/AIDS Knowledge Assessment (HIV-KA)
